# Educational interventions in pharmacovigilance to improve the knowledge, attitude and the report of adverse drug reactions in healthcare professionals: Systematic Review and Meta-analysis

**DOI:** 10.1007/s40199-024-00508-z

**Published:** 2024-03-01

**Authors:** Mónica J. Cervantes-Arellano, Osvaldo D. Castelán-Martínez, Yolanda Marín-Campos, Juan L. Chávez-Pacheco, Olga Morales-Ríos, Laura M. Ubaldo-Reyes

**Affiliations:** 1 Anatomy Department, Facultad de Medicina, Universidad Nacional Autónoma de México (UNAM), Mexico City, Mexico; 2 Clinical Pharmacology Laboratory, UMIEZ, Facultad de Estudios Superiores Zaragoza, Universidad Nacional Autónoma de México (UNAM), Batalla 5 de Mayo s/n Esquina Fuerte de Loreto, Col. Ejército de Oriente, Iztapalapa, Mexico City, C.P. 09230 Mexico; 3 Pharmacology Department, Facultad de Medicina, Universidad Nacional Autónoma de México (UNAM), Mexico City, Mexico; 4 Clinical Pharmacology Laboratory, Instituto Nacional de Pediatría, Mexico City, Mexico; 5 Unidad Habilitada de Apoyo al Predictamen, Hospital Infantil de México Federico Gómez, Mexico City, Mexico

**Keywords:** Pharmacovigilance, Educational interventions, Adverse drug reaction reporting, Systematic review, Meta-analysis

## Abstract

**Objectives:**

Underreporting of adverse drug reactions (ADRs) limits and delays the detection of signs. The aim of this systematic review with meta-analyses was to synthesize the evidence of educational interventions (EIs) efficacy in health professionals to increase ADR reporting, attitudes, and knowledge of pharmacovigilance.

**Evidence acquisition:**

A systematic literature review was carried out to identify randomized clinical trials evaluating the efficacy of EI in pharmacovigilance in health professionals to improve ADR reports, knowledge, and attitude toward pharmacovigilance. ADR reports were pooled by calculating Odds Ratio (OR) with a 95% confidence interval (95%CI), while pharmacovigilance knowledge and attitude were pooled by calculating a mean difference (MD) with 95%CI. In addition, the subanalysis was performed by EI type. Meta-analysis was performed with RevMan 5.4 software. PROSPERO registry CRD42021254270.

**Results:**

Eight hundred seventy-five articles were identified as potentially relevant, and 11 were included in the systematic review. Metanalysis showed that EI increased ADR reporting in comparison with control group (OR = 4.74, [95%CI, 2.46 to 9.12], I^2^ = 93%, 5 studies). In subgroup analysis, the workshops (OR = 6.26, [95%CI, 4.03 to 9.73], I^2^ = 57%, 3 studies) increased ADR reporting more than telephone-based interventions (OR = 2.59, [95%CI, 0.77 to 8.73], I^2^ = 29%, 2 studies) or combined interventions (OR = 5.14, [95%CI, 0.97 to 27.26], I^2^ = 93%, 3 studies). No difference was observed in pharmacovigilance knowledge. However, the subanalysis revealed that workshops increase pharmacovigilance knowledge (SMD = 1.85 [95%CI, 1.44 to 2.27], 1 study). Only one study evaluated ADR reporting attitude among participants and showed a positive effect after the intervention.

**Conclusion:**

EI improves ADR reports and increases pharmacovigilance knowledge. Workshops are the most effective EI to increase ADR reporting.

**Graphical abstract:**

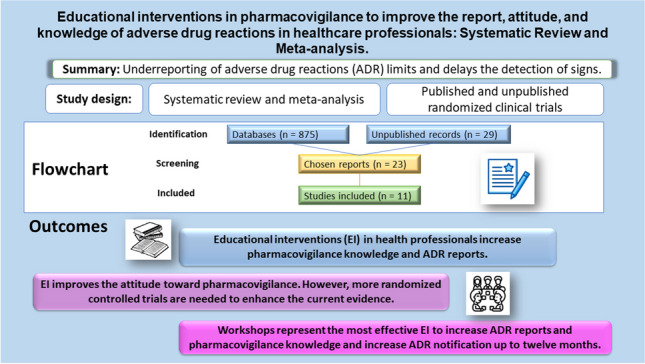

**Supplementary Information:**

The online version contains supplementary material available at 10.1007/s40199-024-00508-z.

## Introduction

Drugs are essential for the treatment of various diseases, but there are drug-related problems, such as adverse drug reactions (ADR) [1]. Post-marketing information on medicines reports a benefit-risk balance obtained from clinical studies. Nevertheless, drug surveillance is necessary to evaluate safety in real-life and long-term conditions [2]. For this reason, ADR voluntary reports are needed, thus spontaneous reporting is the pillar of pharmacovigilance. In countries with pharmacovigilance programs well-established, the report number is about 200 or more per million inhabitants [3]. However, in many countries, pharmacovigilance programs are still under development, and this fact may result in a low drug safety culture that translates into underreporting of ADR. Low notification rates make it difficult to detect signs in the general population that, limits evaluation of ADR causality and the issuance of health alerts. Underreporting can be explained by the low participation of health professionals due to a lack of knowledge and negative attitudes toward pharmacovigilance, such as ignorance (only important serious ADR reports) or lethargy (disinterest in reporting) [3–5].

Different strategies have been evaluated to increase ADR notification, such as the implementation of educational interventions (EI) for health professionals [6–8]. EI purpose is to raise awareness about drug safety issues to improve ADR reporting to obtain statistical assessments by detecting signs and issuing health alerts [9, 10]. Therefore, the aim of this systematic review with meta-analyses was to synthesize the evidence of EI efficacy in health professionals to increase ADR reporting, attitudes, and knowledge of pharmacovigilance.

## Methods

A systematic review and meta-analyses were conducted according to the PRISMA statement (Suppl. 1) [11], and the protocol was prospectively registered in PROSPERO with registration number CRD42021254270.

### Search strategy

A systematic literature search was carried out in the following electronic databases: PubMed, LILACS, Cochrane Central Register of Controlled Trials (CENTRAL), Scopus and Epistemonikos. Unpublished literature was looked up in the abstracts of randomized controlled trials (RCTs) indexed in Scopus Conference Papers and ScienceDirect. Searches were conducted from inception until January 2022 and were not limited by years or language. The strategy search was constructed using the following MeSH terms and keywords: “health personnel”, “physicians”, “pharmacists”, “nurses”; “models educational”, “education medical”; “adverse drug reaction reporting systems”, “pharmacovigilance”, “adverse drug reaction reporting”. The search strategy was adapted to each database (Suppl. 2). In addition, all references identified by systematic reviews were analyzed to identify potentially relevant studies.

### Study selection

Studies were included if they met the following criteria: (1) RCT, including multi-arm trials; (2) participants were health professionals (physicians, consultants, nurses, pharmacists, and dentists); (3) participants received an educational intervention in pharmacovigilance including telephone-based interventions, workshop, educational material, electronic supplementary material, letters, lectures, sessions group, email and combined intervention; in the control group, participants did not receive educational activity or received training from their pharmacovigilance unit; (4) study results were a number of ADR reports and knowledge and attitude mean scores obtained through a questionnaire, in both groups. Studies were excluded if the educational intervention was aimed at patients or if the comparison was made between health professionals and patients, as well as studies that were sponsored by the pharmaceutical industry or involved economic incentives.

 Two independent reviewers (MJC and LMU) assessed all titles and abstracts to identify studies via the inclusion criteria and excluded non-relevant studies. All potentially relevant articles were retrieved and read in full text. Reviewers were blinded to each other’s decisions. Discrepancies were discussed and resolved with a third reviewer (ODC). The inter-rater reliability was evaluated using kappa coefficient.

### Data extraction and risk of bias assessment

Selected studies were reviewed independently by two reviewers (MJC and LMU) to extract in an Excel database the following data: publication year, author, health professionals, EI, time of intervention, control group, the sample size of the intervention group as the control group, participants in both groups, follow-up time, the number of ADR reports, knowledge, and attitude in pharmacovigilance mean score, country, attitude, and knowledge questionnaire (validated or not), change of result over time, ADR type (severe, unexpected, high-causality and new-drugs). Discrepancies in data extraction were resolved by consensus. In case any data was not reported in the article, the authors were contacted to obtain it.

When ADR results were reported in a thousand pharmacist-months, a conversion was made to the number of ADR reports, multiplying the rate per person-month, and dividing by one thousand [12].

Potential biases related to individual RCT were assessed with the Cochrane risk-of-bias tool (RoB 2) [13]. RevMan 5.4 was used to generate the risk of bias figures. [14]. The risk of bias was assessed in duplicates by two authors independently (MJC and LMU). Any disagreement was addressed by reappraisal in conjunction with a third reviewer (ODC).

### Data analysis

Statistical analyses were performed using RevMan 5.4 [14]. ADR reports were pooled using an odds’ ratio (OR) with 95% confidence intervals (95%CI). Knowledge and attitude in pharmacovigilance scores were analyzed with a standardized mean difference (SMD) with 95%CI. All analyses were performed with a random-effects approach. I² test was used to assess the heterogeneity of each evaluate results, and I² > 50% was considered with signification heterogeneity [15]. Subanalysis by type of educational intervention was performed to identify the most effective intervention, as well as to explore heterogeneity between studies. The results’ consistency was evaluated using a leave-one-out sensitivity analysis, the study with the highest bias was excluded in each comparison. Only studies that reported the ADR reports numbers (totals, serious, high probability, unexpected, and new drugs by control and intervention groups before and after the educative intervention), knowledge scores, or changes in attitude were included in the meta-analysis.

## Results

### Characteristics of the studies

**Fig. 1:**
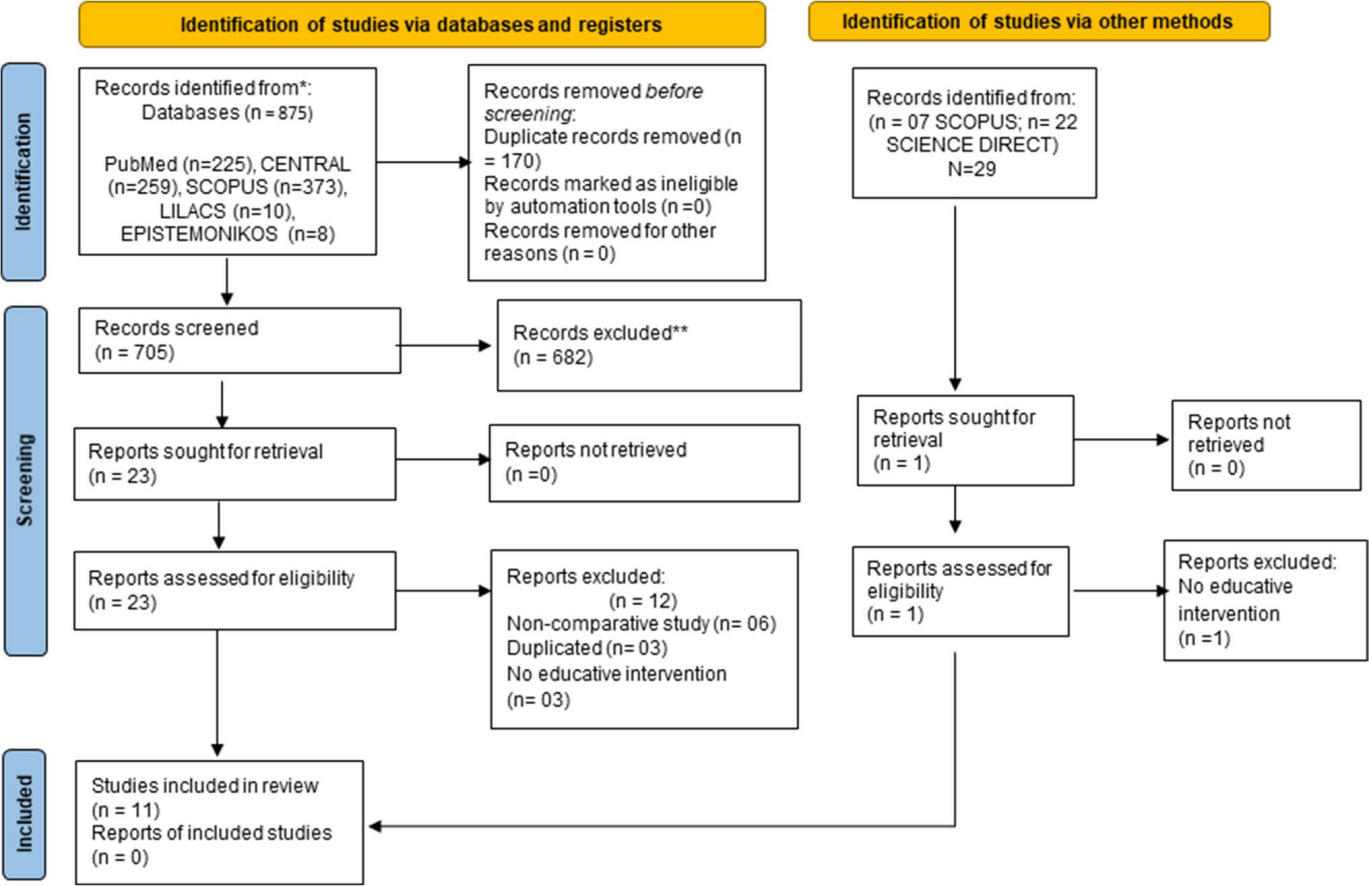
Flowchart for systematic review and meta-analysis (PRISMA) of educational intervention in pharmacovigilance, screening of articles, and selection process

In the systematic search, a total of 875 citations were identified in databases, and the study selection process is illustrated in Fig. 1. After duplicate removal, 705 articles were screened by title and abstract for potential eligibility. In addition, 29 unpublished records were identified. No additional studies were identified in references of previously published systematic reviews. After screening, twenty-four studies were assessed for eligibility, and 13 studies were excluded [16–28]. Non-comparative studies were the main cause of exclusion, and all reasons are shown in the Suppl. 3. Inter-rater agreement was suitable (kappa = 0.83). Eleven studies fulfilled the inclusion criteria of the systematic review, and the characteristics of the included studies are summarized in Table 1. Two authors were contacted for data to be included in the meta-analyses [29, 30], only one responded, however the information could not be pooled. Eight studies were included in the meta-analysis [29, 31–37].

**Table 1:** Characteristics and descriptions of the randomized controlled trials are included in the systematic review

Author (Year)/ country	Health professionals	Educative intervention		Control group		Follow-up (month)	Outcomes*	Questionnaire attitude/ validation
		Intervention	N	Control group	N			
Potlog SM (2020)/ Israel [29]	physicians and nurses	Combined intervention: Program promotion (visiting medical staff), distant learning, lecture, and educational material (posters), for five months.	205	Received no intervention	225	09	The score of “behavior related to reporting” Intervention: Mean ± SD = 2.87 ± 2.37; control: Mean ± SD = 2.48 ± 2.12, *p* = 0.79. The score of “knowledge related to behavior” Intervention: Mean ± SD = 3.67 ± 2.16; control: Mean ± SD = 3.73 ± 2.14, *p* = 0.79.	Yes/No validated
Cheema E (2019)/ Saudi Arabia [37]	pharmacists	Information sheet of ADR and reporting (electronically)	23	Information sheet of coronavirus	23	03	Knowledge score Intervention: Mean ± SD = 7.67 ± 2.1; control: Mean ± SD = 6.71 ± 2.3, *p* = 0.66.	Yes/ No validated
Sarayani A (2015)/ Iran [36]	nurses	1. Lecture: didactic sessions, two sessions of two hours in one day.	143	Received no intervention	212	03	Workshop: Knowledge score = 73.7 ± 11.3 Lecture: Knowledge score = 79.1 ± 11.9	Yes/ No validated
		2. Workshop: brainstorming, two sessions of two hours in one day.	141					
López-González E (2015)/ Spain [35]	physicians	Combined intervention: 1) A group session (25 min) 2) Educational material.	2120	Continuing education course (pharmacovigilance center)	3614	08	ADR total RR = 1.65 [95%CI, 1.08 to 2.53], *p* = 0.021; report of ADR high-causality RR = 1.13 [95%CI, 0.72 to 1.77], *p* = 0.603; report severe ADR = 1.62 [95%CI, 0.99 to 2.65], *p* = 0.056; report unexpected ADR = 2.06 [95%CI, 1.19 to 3.55], *p* = 0.010.	No available
Herdeiro MT (2012)/ Portugal [31]	physicians	1. Telephone intervention: conversation between 3–8 min.	438	Received no intervention	5107	20	1. Telephone interview: ADR total RR = 1.02 [95%CI, 1.00 to 1.04], *p* = 0.052; report of ADR high-causality RR = 0.75 [95%CI, 0.73 to 0.76], *p* < 0.001; report severe ADR = 0.93 [95%CI, 0.91 to 0.94], *p* < 0.001. 2. Workshop: ADR total RR = 3.97 [95%CI, 3.86 to 4.08], *p* < 0.001; report of ADR high-causality RR = 3.58 [95%CI, 3.51 to 3.66], *p* < 0.001; report severe ADR = 6.84 [95%CI, 6.69 to 6.98], *p* < 0.001.	No available
		2. Workshop: one hour.	1034					
Johansson M (2011)/ Sweden [30]	physicians and nurses	Letter: (Information sheet of ADR and reporting, 3 times in 9 months)	77^a^	Received no intervention	74^a^	00	Mean number of reports per unit ± SD = 1.03 ± 2.46, *p* = 0.34; N Total of ADR reports = 79; N ADR reports serious = 12, N unexpected ADR = 20, N new drug-related = 7.	No available
Ribeiro-Vaz I (2011)/ Portugal [34]	pharmacists	1. Telephone intervention: between four and 12 min for 18 days.	261	Received no intervention	1103	20	Report od ADR RR = 3.22 [95%CI, 1.33 to 7.80], *p* = 0.010; report of ADR high level of probability RR = 2.02 [95%CI, 0.74 to 5.49], *p* = 0.168; report severe ADR = 3.87 [95%CI, 1.29 to 11.61], *p* = 0.016; report unexpected ADR RR = 5.02 [95%CI, 1.33 to 18.93], *p* = 0.017.	No available
		2. Workshop: by one month.	103					
Granas AG (2007)/ Norway [38]	pharmacists	Educational material: transparencies, brochures, and posters.	158	Received no intervention.	184	00	Attitude: More positive in the intervention (*p* < 0.001) and more positive in reporting ADR (*p* = 0.01). Report ADR: half (54/105) reported one or more ADRs.	Yes/ No validated
Figueiras A (2006)/ Portugal [33]	physicians	Workshop (visit), reminder card, and report form: two sessions of 30 min.	1388	Received no intervention	5063	13	ADR total RR = 10.23 [95%CI, 3.81 to 27.51], *p* < 0.001; report of ADR high-causality RR = 8.75 [95%CI, 3.05 to 25.07], *p* < 0.001; report severe ADR = 6.32 [95%CI, 2.09 to 19.16], *p* = 0.001; report unexpected ADR = 30.21 [95%CI, 4.54 to 200.84], *p* < 0.001; new drug related ADR = 8.04 [95%CI, 2.10 to 30.83], *p* = 0.002.	No available
Johansson M (2009)/ Sweden [39]	physicians and nurses	E-mails: attached file about new drugs, ADR is important, instructions of the report of ADR (3 times in 9 months)	59^a^	Received no intervention	58^a^	00	N Total of ADR reports = 56, *p* = 0.037; N ADR reports serious = 10, N previously not known ADR = 16, N new drug-related = 4.	No available
Herdeiro MT (2008)/ Portugal [32]	pharmacists	Combined intervention: 1) A group session (30 min of presentation + 30 min of discussion). 2) Educational material on pharmacovigilance for 4 months.	342	Continuing education course (pharmacovigilance center)	1091	12	ADR total RR = 5.87 [95%CI, 1.98 to 17.39], *p* = 0.001; report of ADR high-causality RR = 8.67 [95%CI, 2.12 to 35.42], *p* = 0.002; report severe ADR = 9.79 [95%CI, 2.24 to 42.66], *p* = 0.002; report unexpected ADR = 4.41 [95%CI, 1.11 to 17.53], *p* = 0.04; new drug related ADR = 9.33 [95%CI, 2.53 to 34.40], *p* < 0.001.	No available

N, number ADR report; SD, standard deviation, ADR, Adverse Drug Reaction, 95% CI, Confidence Interval 95%. ^a^Primary Healthcare Units

For country, RCTs were principally conducted in Portugal (four articles) and Sweden (two articles). Geographically, all the studies were conducted in Europe and Asia. The EI varied from one day to nine months, and follow-up ranged from 0 to 20 months. The average participation rate (a healthcare professional who agreed to participate into the study) varies in each study between 7.9 and 84.0%, and participants had more adherence to combined interventions and electronic ADR information.

Four studies involved physicians [29, 31, 33, 35], two involved nurses [29, 36], four involved pharmacists [32, 34, 37, 38], and two studies evaluated primary healthcare units that included physicians and nurses [30, 39]. The professionals mainly studied were physicians (six studies with 5097 participants and 136 primary healthcare units), followed by the pharmacist (four studies with 887 participants) (Table 1).

Workshops were the most common educative interventions used into studies [31, 33, 34, 36], followed by intervention combined (session group and educative material) [29, 32, 35], telephone-based interventions [31, 34], lecture [36], educational material (transparencies, brochures, and posters) [38], electronic information sheet of ADR [37], E-mail interventions [39] and one-page ADR information letter [30]. Three studies included continuing education by the pharmacovigilance unit as a control group [32, 35, 37], while eight studies did nothing [29–34, 36, 38, 39] (Table 1).

### ADR reports

Ten studies informed the number of ADR reports [29–35, 37–39]. Five studies were excluded from the meta-analysis because these have incomplete data such as number of participants, or the total number of ADR reports [29, 30, 37–39]. Five studies present complete data for meta-analysis, and classified ADR as total, serious, high probability, unexpected, and new drugs by control and intervention groups [31–35]. Two studies presented three arms (workshop, telephone-based interventions, and control group) [31, 34], and three studies with two arms (combined intervention or workshop vs. control group) [32, 33, 35].

**Fig. 2:**
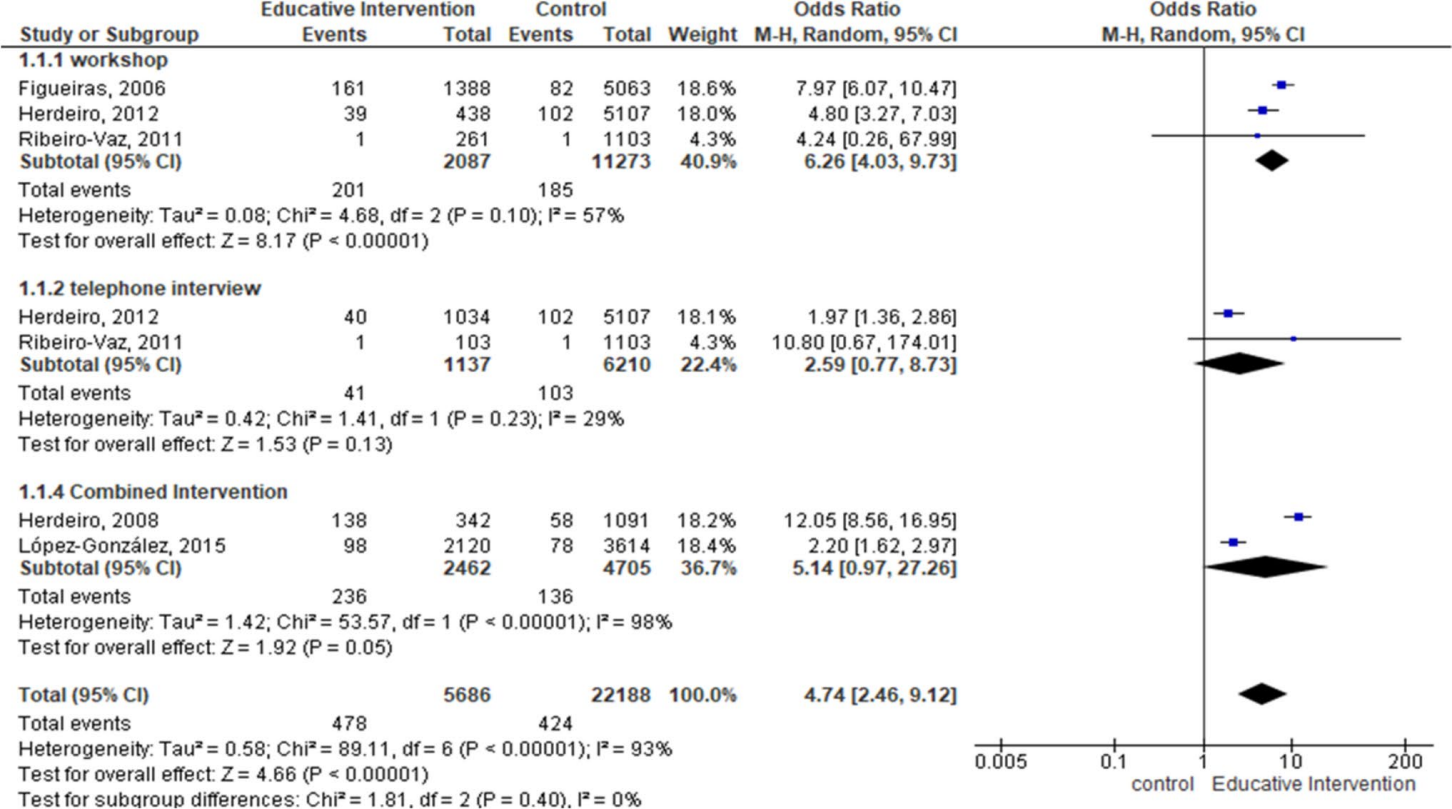
Forest plot of total adverse drug reactions reported for each educational intervention at the end of the study. Sub-analysis was performed by type of intervention

Educational interventions increased the reporting of all ADRs in comparison with control group (OR = 4.74, [95%CI, 2.46 to 9.12], I^2^ = 93%, 5 studies). In the sensitivity analysis, after removed Herdeiro et al. [31], educational interventions showed consistency in increasing ADR reporting (OR = 6.06 [95%CI, 2.50 to 14.71], I^2^ = 94%, 4 studies). In subgroup analysis, workshops (OR = 6.26, [95%CI, 4.03 to 9.73], I^2^ = 57%, 3 studies) increased ADR reporting, more than combined interventions (OR = 5.14, [95%CI, 0.97 to 27.26], I^2^ = 98%, 3 studies), while telephone-based interventions no showed a difference (OR = 2.59, [95%CI, 0.77 to 8.73], I^2^ = 29%, 2 studies) (Figs. 2).

ADR reporting change over time is shown in Table 2. In the workshop intervention, the increase in the number of reports was significant up to 16 months after IE for total and severe ADRs, but only increased over 12 months for unexpected, high-causality, and new drug ADRs. In contrast, telephone-based interventions only increased the number of total reports and serious ADRs by 4 months. Interestingly, the combined interventions increased the number of unexpected and new drug ADRs for at least 12 months, although for total, serious, and high-causality ADRs, the effect was seen from 12 months onwards.

**Table 2:** Report of the total, serious, high-causality, unexpected, and new drugs ADRs over time, once the application of the intervention has ended

Follow-up, months	4	8	12	16	4	8	12	16	4	8	12	16
Intervention	Workshop [31, 33, 34]				Telephone interview [31, 34]				Combined intervention: Session group and educative material [29, 32, 35]			
Comparator	No intervention								No intervention/continuing course			
OR (95% IC), p												
Total	**17.0 (11.6–25.1), 0.00**	**4.0 (2.5–6.6), 0.00**	**3.5 (2.2–5.7), 0.00**	**3.1 (1.3–7.5), 0.01**	**4.3 (2.0-9.3), 0.00**	1.7 (0.7–3.9), 0.25	2.5 (0.9–6.6), 0.07	0.7 (0.3–1.8), 0.46	**8.48 (0.8–88.1), 0.007**	**3.1 (0.9–11.2), 0.08**	**4.0 (2.3–6.9), 0.00**	**7.3 (2.3–24.0), 0.00**
Serious	**8.7 (5.1–14.8), 0.00**	**1.4 (0.6–3.6), 0.46**	**4.1 (2.2–7.9), 0.00**	**2.6 (1.2–6.1), 0.02**	**5.0 (2.0-12.6), 0.00**	2.5 (0.7–8.2), 0.14	1.8 (0.6–5.7), 0.32	0.8 (0.2–2.6), 0.69	9.8 (0.7-137.1), 0.09	3.2 (0.6–18.1), 0.19	**3.3 (1.5–7.4), 0.00**	**55.5 (3.2-963.6), 0.01**
High causality	**13.6 (8.9–20.7), 0.00**	**3.9 (2.2-7.0), 0.00**	**3.3 (2.0-5.7), 0.00**	2.7 (0.9–8.3), 0.08	2.5 (0.9–6.6), 0.07	2.2 (0.9–5.3), 0.09	2.8 (0.9–8.2), 0.07	0.5 (0.2–1.6), 0.22	7.6 (0.8–73.4), 0.08	3.6 (0.4–31.9), 0.25	**3.9 (2.0-7.6), 0.00**	**7.6 (2.0-29.5), 0.00**
Unexpected	**108.0 (13.8-846.6), 0.00**	**3.6 (1.2–10.7), 0.02**	**7.0 (2.5–20.0), 0.00**	5.6 (0.4–82.2), 0.21	14.4 (0.6-364.2), 0.10	3.0 (0.7–12.4), 0.14	1.7 (0.2–15.9), 0.67	0.6 (0.1–4.9), 0.65	**7.4 (2.0-26.9), 0.00**	**3.2 (1.4–7.3), 0.01**	**4.3 (1.5–12.6), 0.01**	0.6 (0.0-13.3), 0.77
New drugs	**10.9 (4.2–28.3), 0.00**	**3.5 (1.2–10.4), 0.02**	**4.8 (2.3–10.0), 0.00**	3.2 (0.29–36.2), 0.34	2.5 (0.7–8.2), 0.14	0.4 (0.1–2.7), 0.31	1.0 (0.1–8.5), 0.99	0.2 (0.0-2.9), 0.22	**20.51 (7.06–59.55), 0.00**	**8.1 (3.3–19.7), 0.00**	**3.6 (1.5-9.0), 0.01**	**9.7 (2.0-48.4), 0.01**

*N *total studies, *OR *odds ratio, *95% IC* 95% confidence intervals, *p* p-value, 4: four months after ending educational intervention, 8: eight months after ending educational intervention, 12: twelve months after ending educational intervention, 16: sixteen months after ending educational intervention. Statistically significant results are shown in bold

### Knowledge, and attitude in pharmacovigilance

**Fig. 3:**
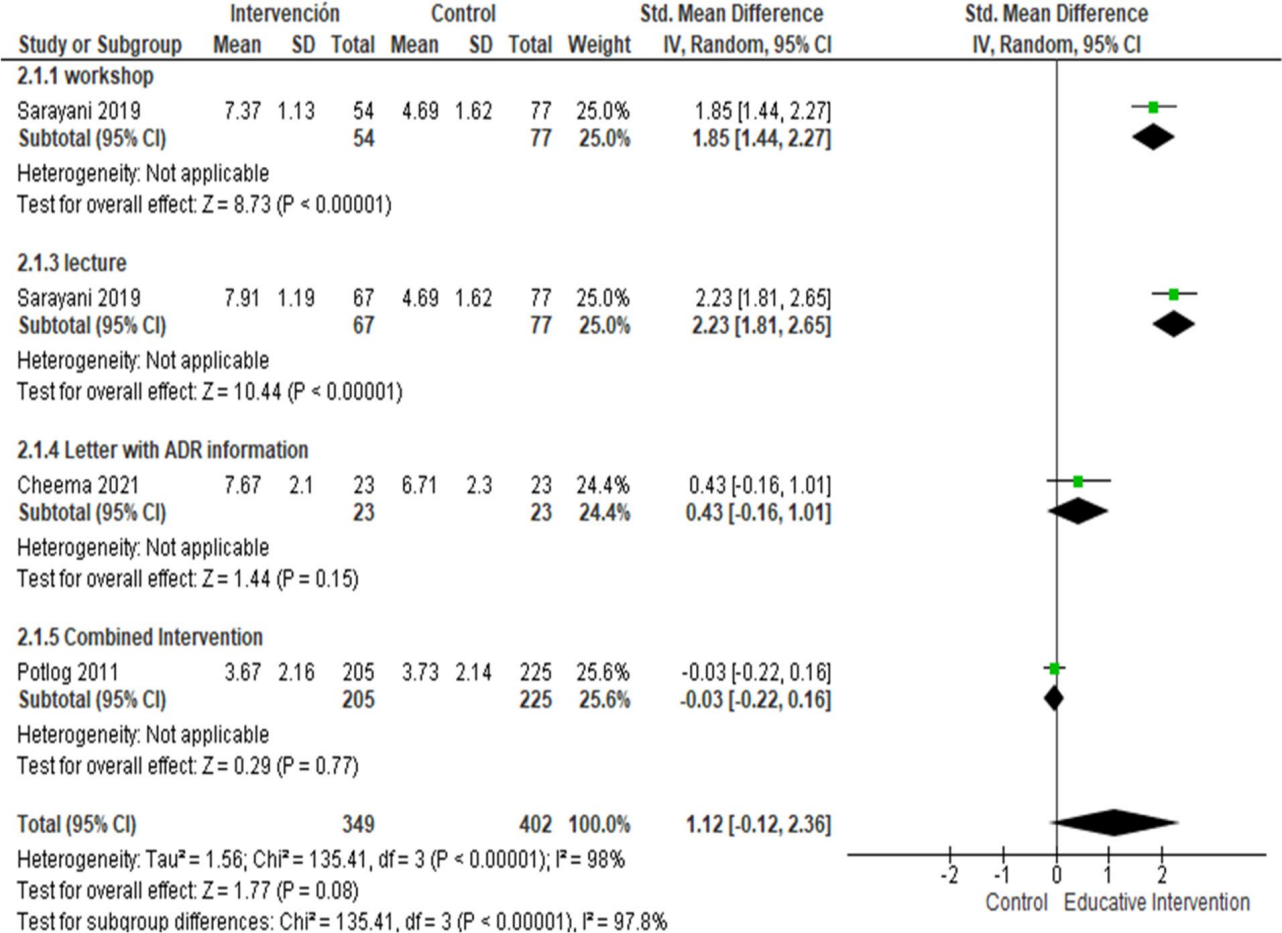
Forest plot of the difference in means of the effect of EI in the score of knowledge about pharmacovigilance at the end of the study. Sub-analysis was performed by type of intervention

Regarding the change in knowledge in pharmacovigilance, three studies [29, 36, 37] evaluated 4 educative interventions. The meta-analysis results showed a tendency to increase pharmacovigilance knowledge mean scores in participants who received EI in comparison with the control group (SMD = 1.12, [95%CI, -0.12 to 2.36], I^2^ = 98%, 4 studies). After removing the highest risk of bias study [29], participants in EI group shown an augmented their pharmacovigilance knowledge (SMD = 1.53 [95%CI, 0.58 to 2.47, I^2^ = 92%, 3 studies]). In subgroup analysis, the participants who received lecture (SMD = 2.23 [95%CI, 1.81 to 2.65], 1 study) and workshop (SMD = 1.85 [95%CI, 1.44 to 2.27], 1 study) increased their knowledge; this effect was not observed in those who received the combined intervention or letter with ADR information (Fig. 3).

Two studies evaluated ADR reporting attitudes among health professionals (Table 1), however, the measurement scales obtained by the questionnaire are different, so it was not possible to perform a meta-analysis. One study conducted in pharmacist showed a positive attitude toward ADR reporting after the intervention [38]. Likewise, a positive effect in behavior related to reporting was observed in physicians and nurses after educative intervention [29].

### Risk of bias assessment

**Fig. 4:**
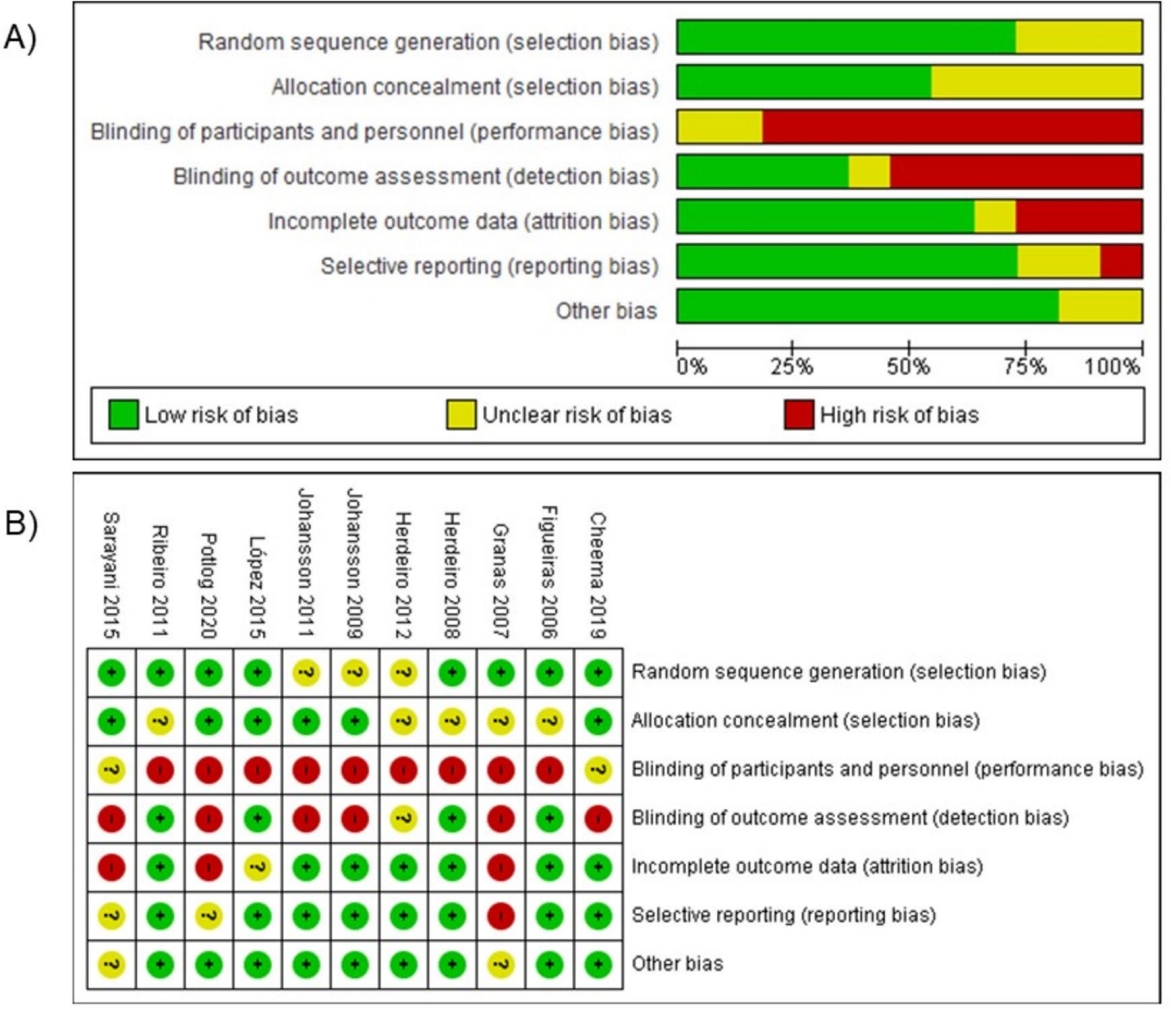
Risk of bias graph, review authors’ judgments about each risk of bias item presented as percentages across all included studies. **A** Risk of bias overall assessment, the proportion of assessment studies. **B** Risk of bias assessment summary for included studies

In risk of bias assessment (Fig. 4), 73% of studies had adequate random sequence generation [29, 32–38]. Only 54% describe the randomization process completely [29, 30, 35–37, 39], presenting low-risk allocation concealment, because the randomization was carried out by a person outside the study, or they avoided contamination between groups by randomizing health centers.

The performance bias had a high risk in at least 81% of articles, due to differences in interventions ranging from a phone call to a combined intervention [29–35, 38, 39]. With respect to blinding outcome assessment, in 4 studies the ADR reports evaluator was blinding [32–35]. In 63% of the studies [30–34, 37, 39], no missing data were seen, while reporting bias was considered a low risk in 72% of studies [30–35, 37, 39]. Additionally, in other potential sources of bias, 80% (9 of 11 studies) of the selected studies were rated with a low risk of bias [29–34, 37, 39].

## Discussion

ADR report is paramount for causality analysis and drug safety assessment. Nonetheless, ADR occurrence generates distrust in health professionals due to the fear of being judged and punished [40]. To avoid this, EIs in pharmacovigilance are intended to increase knowledge about drug safety, improve attitudes towards ADRs, and consequently increase the reporting. The results of this systematic review with meta-analysis showed that EI in pharmacovigilance increases the ADR reports, and present positive changes in pharmacovigilance knowledge and attitude in health care professionals.

To synthesize the best available evidence on the role of EI in increasing ADR reporting, only RCTs were included in this systematic review. Study results show that EI increases by about four times the ADR report. Similar results were reported in a systematic review that synthesized the evidence on interventions to increase the spontaneous reporting of ADRs in healthcare professionals and patients [8]. Likewise, two previous systematic reviews, which included pre-post experimental design, quasi-experimental and RCT studies, concluded that the interventions evaluated were considered effective [6, 8]. However, no previous systematic review has evaluated efficacy by intervention type. In this study, the workshops have greater ADR reporting efficacy compared to others, that could be explained by the person-person interaction of the workshop allows a better understanding of the concept compared to reading information in a letter. In this sense, the score of knowledge observed in workshop participants is two-fold increase in comparison with participants who received a letter with an ADR information. Previous results indicated that interactive sessions enhance participant activity and provide the opportunity to practice skills can effect change in professional practice [41].

In addition, the effectiveness over time reveals that EI with interaction between people such as workshops and combined interventions maintain their effect on the ADR report for up to 16 months. This effect was not observed in telephone-based intervention, it suggests the necessity for a re-intervention.

Furthermore, educational combined intervention can reinforce and increase the understanding of pharmacovigilance issues and modify the attitude about ADR and increase the report in comparison with a simple intervention [7, 42]. Similarly, Forsetlund L., recommends using combined interventions with interactive formats that increase attention, to increase the effectiveness of the interventions [43]. It is not certain that printed educational materials, as a single intervention, can maintain the change in results over time [44]. In contrast, regular delivery of drug safety information can be an effective and inexpensive technique, but it loses its effect if delivery is stopped [45]. In this systematic review, all the studies that evaluated the combined intervention used the continuing education of the pharmacovigilance unit as a comparator. This could explain why, although there is a trend in favor of the combined intervention for the increase in the total ADR reports, this is not statistically significant.

The educative interventions dependent on complex factors such as intrapersonal, interpersonal, professional education, context, and material quality [41]. The educational intervention could work depending on the population, the objective sought, and due to the training of the participant. In RCTs included in this systematic review have no harmonization in the type of educational intervention and length. In this way, EI investigated in pharmacovigilance are different, regardless of the study design, and have durations ranging from a few minutes to six years [7]. These differences can be explained by cultural gaps, and social situations in each region that could modify the intervention type according to the context of each country, such as the geographical location and status of the pharmacovigilance system [23]. EI explored into the studies included in this systematic review were evaluated in Europe and Asia countries, appraisal of these interventions in other countries using RCTs approach may provide information on the efficacy of EI in regions whose drug safety culture may be different.

In clinical practice, the effectiveness of EI in pharmacovigilance can be increased by existence of continuous training in the study, reporting promotion by regional centers, the unit’s requirement to report cases of a new drug, an industry study, incentive programs for reporting, electronic methods of ADR report, and monetary incentives [6, 42, 46, 47]. Against, the effectiveness can be decreased due to factors such as high workload that does not allow reporting, limited time to take courses and lack of interest in pharmacovigilance [45, 48]. In this sense, the attitude to ADR underreporting can be explained by Inman and its seven deadly sins: complacency, ignorance, diffidence, financial incentives, legal aspects, lethargy, and indifference [4]. Furthermore, the fact that health professionals have a high knowledge of pharmacovigilance does not imply that they have a good attitude towards the report [49, 50]. Previous studies based on questionnaires of Knowledge, Attitude, and Practice (KAP) in pharmacovigilance support that an educational intervention could generate a change in a positive behavior on ADR report [6, 50–53]. Only one RCT in this systematic review evaluated attitude after educative intervention, with a positive effect [38].

ADR reporting in post-marketing surveillance is a cornerstone for signal detection and contribute to establish guidelines or policies for medication use. Consequently, it allows identifying serious or unexpected adverse drug reactions that represent a major problem in patient safety and increase hospital costs; thus, educative interventions sensitize health professionals about its importance [54]. In this review, the workshops and combined interventions increase the serious, unexpected, high causality, and new drug ADR reporting for at least 12 months.

### Limitation of study

This systematic review has the following limitations, which should be considered when interpreting the results: (1) the educational interventions are different, such as workshops, combined interventions, telephone-based interventions, letters, or lectures; (2) the studies were evaluated with two different types of controls (continuing education and nothing); (3) No study that evaluated knowledge or attitude performed a prior validation of the questionnaire; (4) the workshop variate between brainstorming with two sessions of two hours in one day, one session of one hour, a session every month, or reminder card and report form with two sessions of 30 min.

## Conclusions

The educative interventions in pharmacovigilance increased the number of ADR reports and score in the knowledge. The workshop and combined intervention are the EI with greater efficacy and duration. More RCTs are needed to assess the role of educational interventions in changing attitudes towards pharmacovigilance.

### Supplementary information

Below is the link to the electronic supplementary material.

ESM 1 (DOCX 21.3 KB)

ESM 2 (DOCX 16.0 KB)

ESM 3 (DOCX 14.1 KB)

## Data Availability

Databases generated for this systematic review are available from the corresponding author upon reasonable request.
