# Supplementary material for: Educational interventions in pharmacovigilance to improve the knowledge, attitude and the report of adverse drug reactions in healthcare professionals: Systematic Review and Meta-analysis
Source: Daru. 2024 Mar 1;32(1):421–34. doi: 10.1007/s40199-024-00508-z (PMC11087385; doi:10.1007/s40199-024-00508-z)
Supplement: Supplementary file 2 — (DOCX 16.0 KB) [file 40199_2024_508_MOESM2_ESM.docx]

Supplementary 2. Search strategy in the electronic databases.

| Database | Search algorithm | Publications numbers | Date, hour |
| --- | --- | --- | --- |
| PubMed | ("Health Personnel"[Mesh] OR "Physicians"[Mesh] OR "Physical Therapists"[Mesh] OR "Surgeons"[Mesh] OR "Pharmacists"[Mesh] OR "Nurses"[Mesh] OR "Caregivers"[Mesh] OR "Nursing Assistants"[Mesh] OR "Dentists"[Mesh] OR "Orthodontists"[Mesh] OR "Oral and Maxillofacial Surgeons"[Mesh] OR "Endodontists"[Mesh] OR ”Health professional”[TIAB] OR “Health care professional”[TIAB] OR “healthcare professional”[TIAB] OR “Physicians”[TIAB] OR “Doctor”[TIAB] OR “Medical specialist”[TIAB] OR “Specialist physician”[TIAB] OR “Specialist doctor”[TIAB] OR “Medical person”[TIAB] OR “Surgeon”[TIAB] OR “Pharmacist”[TIAB] OR “Pharmacy”[TIAB] OR “Nurses”[TIAB] OR “Caregiver”[TIAB] OR “Assistant”[TIAB] OR “Attendant”[TIAB] OR “Therapist”[TIAB] OR “Medic”[TIAB] OR “Registered nurse”[TIAB] OR “Caretaker”[TIAB] OR “Nursing”[TIAB] OR “Dentist”[TIAB] OR “Prosthodontist”[TIAB] OR “Endodontist”[TIAB] OR “Exodontist”[TIAB] OR “Orthodontist”[TIAB]) AND ("Models, Educational"[Mesh] OR "Education, Medical"[Mesh] OR "Education, Medical, Graduate"[Mesh] OR "Electronic Mail"[Mesh] OR “Educational strategy”[TIAB] OR “Education strategies”[TIAB] OR “Education, medical”[TIAB] OR “Educational intervention”[TIAB]OR “Telephone interviews”[TIAB] OR “workshops”[TIAB] OR “outreach visit”[TIAB] OR “reminder card”[TIAB] OR “form report”[TIAB] OR “yellow card placement”[TIAB] OR “slide presentation”[TIAB] OR “distribution of press material leaflets”[TIAB] OR “e-mail”[TIAB] OR “one-page ADR information letter”[TIAB] OR “Health Knowledge, Attitudes, Practice"[Mesh]) AND ("Adverse Drug Reaction Reporting Systems"[Mesh] OR "Pharmacovigilance"[Mesh] OR “Adverse drug reaction reporting”[TIAB] OR “Adverse drug reported spontaneously”[TIAB] OR “Adverse drug reaction reporting systems”[TIAB] OR “Pharmacovigilance” [TIAB]) | 225 | 01/23/2022  22:35 |
| CENTRAL | "Health Personnel" OR "Physicians" OR "Physical Therapists" OR "Surgeons" OR "Pharmacists" OR "Nurses" OR "Caregivers" OR "Nursing Assistants" OR "Dentists" OR "Orthodontists" OR "Oral and Maxillofacial Surgeons" OR "Endodontists" OR "health professional" OR "health care professional" OR "healthcare professional" OR "Doctor" OR "medical specialist" OR "specialist physician" OR "specialist doctor" OR "medical person" OR "Pharmacy" OR "Assistant" OR "Attendant" OR "Therapist" OR "Medic" OR "Registered nurse" OR "Caretaker" OR "Nursing" OR "Prosthodontist" OR "Exodontist" OR "Orthodontist"  N=131,763  "Models, Educational" OR "Education, Medical" OR "Education, Medical, Graduate" OR "Electronic Mail” OR "educational strategy" OR "education strategies" OR "educational intervention" OR "Telephone interviews" OR "workshops" OR "outreach visit" OR "reminder card" OR "form report" OR "yellow card placement" OR "slide presentation" OR "distribution of press material leaflets" OR "e-mail" OR "one-page ADR information letter" OR “Health Knowledge, Attitudes, Practice"  N= 0  "Adverse Drug Reaction Reporting Systems" OR "Pharmacovigilance" OR "Adverse drug reaction reporting" OR "Adverse drug reported spontaneously"  N=590 | 259 | 01/23/2022  22:37 |
| SCOPUS | TITLE-ABS-KEY("Health Personnel" OR "Physicians" OR "Physical Therapists" OR "Surgeons" OR "Pharmacists" OR "Nurses" OR "Caregivers" OR "Nursing Assistants" OR "Dentists" OR "Orthodontists" OR "Oral and Maxillofacial Surgeons" OR "Endodontists" OR "health professional" OR "health care professional" OR "healthcare professional" OR "Doctor" OR "medical specialist" OR "specialist physician" OR "specialist doctor" OR "medical person" OR "Pharmacy" OR "Assistant" OR "Attendant" OR "Therapist" OR "Medic" OR "Registered nurse" OR "Caretaker" OR "Nursing" OR "Prosthodontist" OR "Exodontist" OR "Orthodontist") AND TITLE-ABS-KEY("Models, Educational" OR "Education, Medical" OR "Education, Medical, Graduate" OR "Electronic Mail" OR "educational strategy" OR "education strategies" OR "educational intervention" OR "Telephone interviews" OR "workshops" OR "outreach visit" OR "reminder card" OR "form report" OR "yellow card placement" OR "slide presentation" OR "distribution of press material leaflets" OR "e-mail" OR "one-page ADR information letter") OR “Health Knowledge, Attitudes, Practice" AND TITLE-ABS-KEY("Adverse Drug Reaction Reporting Systems" OR "Pharmacovigilance" OR "Adverse drug reaction reporting" OR "Adverse drug reported spontaneously") | 373 | 01/23/2022  22:40 |
| LILACS | Educational Pharmacovigilance | 10 | 01/23/2022  22:42 |
| Epistemonikos | (“Educational intervention”) AND ("Pharmacovigilance") | 08 | 01/23/2022  22:44 |
| Science Direct | (“Educational intervention”) AND ("Pharmacovigilance") | 22 | 01/23/2022  22:45 |
| SCOPUS Conference papers | TITLE-ABS-KEY("Health Personnel" OR "Physicians" OR "Physical Therapists" OR "Surgeons" OR "Pharmacists" OR "Nurses" OR "Caregivers" OR "Nursing Assistants" OR "Dentists" OR "Orthodontists" OR "Oral and Maxillofacial Surgeons" OR "Endodontists" OR "health professional" OR "health care professional" OR "healthcare professional" OR "Doctor" OR "medical specialist" OR "specialist physician" OR "specialist doctor" OR "medical person" OR "Pharmacy" OR "Assistant" OR "Attendant" OR "Therapist" OR "Medic" OR "Registered nurse" OR "Caretaker" OR "Nursing" OR "Prosthodontist" OR "Exodontist" OR "Orthodontist") AND TITLE-ABS-KEY("Models, Educational" OR "Education, Medical" OR "Education, Medical, Graduate" OR "Electronic Mail" OR "educational strategy" OR "education strategies" OR "educational intervention" OR "Telephone interviews" OR "workshops" OR "outreach visit" OR "reminder card" OR "form report" OR "yellow card placement" OR "slide presentation" OR "distribution of press material leaflets" OR "e-mail" OR "one-page ADR information letter") OR “Health Knowledge, Attitudes, Practice" AND TITLE-ABS-KEY("Adverse Drug Reaction Reporting Systems" OR "Pharmacovigilance" OR "Adverse drug reaction reporting" OR "Adverse drug reported spontaneously") | 07 | 01/23/2022  22:50 |
