# Supplementary material for: Educational interventions in pharmacovigilance to improve the knowledge, attitude and the report of adverse drug reactions in healthcare professionals: Systematic Review and Meta-analysis
Source: Daru. 2024 Mar 1;32(1):421–34. doi: 10.1007/s40199-024-00508-z (PMC11087385; doi:10.1007/s40199-024-00508-z)
Supplement: Supplementary file 3 — (DOCX 14.1 KB) [file 40199_2024_508_MOESM3_ESM.docx]

Supplementary 3. Articles excluded after selection for inclusion criteria.

| AUTOR, YEAR | REASON FOR EXCLUSION |
| --- | --- |
| Bäckström, 2006 | No educative intervention |
| Bracchi, 2005 | Noncomparative study |
| Kane-Gill, 2016 | No educative intervention |
| Stoynova, 2013 | Noncomparative study |
| Gonzalez -Gonzalez, 2013 | Population duplicated |
| Sanghavi, 2013 | Noncomparative study |
| Ribeiro, 2009 | Population duplicated |
| Rosenbaum, 1992 | Noncomparative study |
| Herdeiro, 2012 | Population duplicated |
| Jha, 2014 | Noncomparative study |
| Srikanth, 2018 | Noncomparative study |
| Williams, 2017 | No educative intervention |
| Gumustekin, 2017 | No educative intervention |
